# Interaction of nanoparticles with proteins: relation to bio-reactivity of the nanoparticle

**DOI:** 10.1186/1477-3155-11-26

**Published:** 2013-07-19

**Authors:** Shruti R Saptarshi, Albert Duschl, Andreas L Lopata

**Affiliations:** 1 Centre for Biodiscovery and Molecular Development of Therapeutics, School of Pharmacy and Molecular Science, James Cook University, Townsville, Queensland, Australia; 2 Department of Molecular Biology, University of Salzburg, Salzburg, Austria

**Keywords:** Nanoparticle, Protein corona, Protein unfolding, Nanoparticle uptake, Circular dichroism

## Abstract

Interaction of nanoparticles with proteins is the basis of nanoparticle bio-reactivity. This interaction gives rise to the formation of a dynamic nanoparticle-protein corona. The protein corona may influence cellular uptake, inflammation, accumulation, degradation and clearance of the nanoparticles. Furthermore, the nanoparticle surface can induce conformational changes in adsorbed protein molecules which may affect the overall bio-reactivity of the nanoparticle. In depth understanding of such interactions can be directed towards generating bio-compatible nanomaterials with controlled surface characteristics in a biological environment. The main aim of this review is to summarise current knowledge on factors that influence nanoparticle-protein interactions and their implications on cellular uptake.

## General introduction

Nanoparticles (NPs) have unique properties that may be useful in a diverse range of applications, and consequently they have attracted significant interest. Particularly in the bio-medical field, the use of nano vaccines and nano drugs are being intensively researched. Nevertheless, our knowledge about the bio-compatibility and risks of exposure to nanomaterials is limited. Exposure to nanomaterials for humans may be accidental, for example occupational exposure, or intentional, for example in the use of nano-enabled consumer products. There are an increasing number of studies that demonstrate adverse effects of nanomaterials in *in-vitro* cellular systems, but it is unclear whether the available data can be reliably extrapolated to predict the adverse effects of nanotechnology for humans. Hence, there is an urgent need to understand the molecular mechanisms of nanoparticles-to-biological system interaction.

In a biological medium, NPs may interact with bio-molecules such as proteins, nucleic acids, lipids and even biological metabolites due to their nano-size and large surface-to-mass ratio. Of particular importance is the adsorption of proteins on the nanoparticle surface. The formation of nanoparticle-protein complexes is commonly referred to as the nanoparticle-protein corona (NP-PC). A number of consequences of protein adsorption on the NP surface can be speculated. Overall, the NP-PC can influence the biological reactivity of the NP [1,2].

This review gives a summary of the current research on the physico-chemical characteristics influencing the formation of the NP-PC, its impact on the structure of adsorbed proteins and the overall implication these interactions have on cellular functions.

### Nanoparticle protein corona

Proteins are polypeptides with a defined conformation and carry a net surface charge depending on the pH of the surrounding medium. Adsorption of proteins at the nano-bio interface is aided by several forces such as hydrogen bonds, solvation forces, Van der Waals interactions, etc. The overall NP-PC formation is a multifactorial process and not only depends on the characteristics of the NP, but also on the interacting proteins and the medium. Specific association and dissociation rates for each protein decide longevity of their interaction with the NP surface. Irreversible (or at least long-term) binding of proteins on the NP leads to formation of a “hard corona” whereas quick reversible binding of proteins that have faster exchange rates defines a “soft corona” [2-6].

Serum/plasma cellular proteins represent complex biological systems, and it has to be considered that NPs can form Bio/Nano complexes when exposed to several, very different systems *in vivo*. An inhaled NP may pass through the mucosal layer, lung epithelial cells and finally enter in to the blood. Similarly, at the cellular level after being phagocytised by a monocyte, the NP may be taken into the endosomes that ultimately fuse with lysosomes. Each of these proteomes represents unique environments and has specific properties with respect to their protein composition, enzymatic activities, pH, ion composition etc. These environments may cause the NP to undergo a complex sequence of modifications that are far from being fully understood. Even within one environment the NP-protein interactions are constantly changing. For example, when exposed to blood plasma the nano-bio interface has been reported to change with time due to constant adsorption and desorption of proteins [1]. NPs that have entered the body thus have to be considered as evolving systems that are shaped by sequential exposure to different protein rich environments. Kinetics of protein adsorption on the NP surface can be influenced by several factors. Amount of proteins available to interact with the NP surface is one such factor that can greatly influence the NP-PC composition. When plasma proteins were applied at concentrations between 3% and 80% of plasma, Monopoli and co-workers observed that the proteins bound to NPs varied with plasma concentration, while relative amounts of some abundant proteins adsorbed on surfaces of silica or polystyrene NPs increased with increasing plasma concentrations [7]. When travelling through different protein rich environments in an *in vivo* system the NP surface may get pre-coated with specific proteins. This can also determine which new protein will bind to the already formed NP-protein complex. Pre-coating of pulmonary surfactant proteins was shown to influence the subsequent adsorption of plasma proteins on the surface of multi walled carbon nanotubes (MWCNT) [8]. Also, silica or polystyrene NPs were shown to retain a “fingerprint” of plasma proteins even after subsequent incubations with other biological fluids [9].

In human plasma, a typical NP-PC consists of proteins like serum albumin, immunoglobulins, fibrinogen, apolipoproteins etc (Table 1). A recent study by Hellstrand and co-workers showed the presence of high density lipoproteins in the protein corona on polystyrene NPs [10]. The adsorption pattern of blood proteins to foreign inorganic surfaces is dynamic where more abundant proteins such as albumin and fibrinogen may initially occupy the surface and get subsequently replaced by other proteins having higher binding affinity for the surface. Such a sequential binding pattern of plasma proteins is based on the Vroman [11] theory and has also been suggested for nano-surfaces. The order of plasma protein binding to single walled carbon nanotubes (SWCNT) was fibrinogen followed by immunoglobulin, transferrin and albumin [12]. Displacement of albumin by other cell lysate proteins was demonstrated for nanomaterials investigated by Sund and co-workers [13]. By contrast, plasma protein binding to ultra-small super paramagnetic iron oxide (SPION) nanoparticle surface did not follow the Vroman theory when exposed to plasma proteins [14]. Therefore, displacement of proteins with time is not a universal rule that can be taken for granted for all types of NPs.

**Table 1:** Comprehensive overview of serum/plasma proteins adsorbed on the surface of different types of nanomaterials with varied size and surface chemistries

**Nanomaterial used**	**Size (nm)**	**Surface chemistry**	**Dispersal medium**	**Proteins identified**	**Ref**
Polystyrene NPs	50, 100	NH_2_, COOH	Human Plasma	Coagulation factors, Immunoglobulins, Lipoproteins, Complement proteins, Acute phase proteins	[15]
Polystyrene NPs	100	COOH	Human serum (depleted)	Complement proteins, Plasminogen, Anti-CD4, c4a, Immunoglobulin, Albumin, Complement , Plasminogen	[16]
Latex NPs	80-109	NH_2_,NHR, NR_2_,NR_3_^+^ COO^-^, SO^−^_3_, SO^−^_4_	Human Serum	Albumin, Apolipoproteins, Immunoglobulins, Hemoglobin, Haptoglobins	[17]
Copolymer NPs	70, 200	-	Human Plasma	Albumin, Apolipoprotiens, Fibrinogen, Immunoglobulins, C4BPαchain	[2]
MWCNTs	20-30	NH_2_, COOH	Human Plasma	2 Macroglobulin precursor, Complement factors, plasminogen, Coagulation factors	[8]
SPIONs	-	-	Human Plasma	Albumin, α1Antitrypsin, Fibrinogen chains, Immunoglobulin chains, Transferrin, Transthyretin	[14]
Gold	5, 10, 20	(PAA) polymer coated	Human Plasma	Albumin, Fibrinogen chains, Apolipoprotein A1	[18]
Gold	15, 40, 80	-	Bovine Serum	Transport proteins, Coagulation factors, Tissue development proteins	[19]
TiO_2_ NPs ZnO NPs Quartz sand Carbon nanotubes SiO_2_ NPs	-	Silone, alumina, silica coated	Human Plasma	Fibrinogen chains, Immunoglobulin light chains, Fibrin, albumin, ApoA1, Complement component proteins, Fibronectin,	[13]
SiO_2_ NPs	8, 20, 25	-	Human Plasma	Immunoglobulins, Lipoproteins, Complement proteins, Coagulation proteins, Acute phase proteins, Cellular proteins, Serum proteins	[20]
TiO_2_ NPs ZnO NPs SiO_2_ NPs	-	-	Human Plasma	Albumin, Immunoglobulins, Fibrinogen, Transferrin, Apolipoprotein A1,Complement proteins	[21]
Magnetic NPs	50, 200	Dextran COOH, NH_2_,PEG COOH, PEG-NH_2_	Bovine Serum	Albumin, Apolipoprotein A-1 Complement factors, Vitronectin, Haemoglobin	[22]

Adsorption of a protein on the NP surface also depends on the affinity of the protein towards the NP surface and its ability to completely occupy the surface. The way in which protein molecules arrange themselves on the NP surface may affect the biological reactivity of the latter at the cellular level [12]. Plasma proteins such as human serum albumin (HSA) and transferrin were shown to adsorb in a monolayer fashion on iron-platinum (FePt) NP surface [23]. Rezwan et al. observed that bovine serum albumin (BSA) adsorbs on aluminium oxide surface as a monolayer by using 30-36% of its total negative charge and that additional BSA molecules from the medium bind onto this monolayer as dimers [24].Detailed studies along these lines can be useful in designing protein-conjugated NP surfaces for future applications.

### Nanoparticles induce changes in the structure of adsorbed proteins

The NP surface can modify the structure and therefore the function of the adsorbed protein thus affecting the overall bio-reactivity of the NP. This section further explores the fate of the proteins bound on the NP surface. Curved NP surfaces compared to planar surfaces provide extra flexibility and enhanced surface area to the adsorbed protein molecules [25]. Curved NP surfaces can also affect the secondary structures of proteins, and in some cases cause irreversible changes [26]. It is interesting to note that chemical properties of individual proteins and their structural flexibility also play an important role in regulating such surface-driven modifications to their secondary structures [27]. Gold NPs were shown to influence conformational changes in the structure of bovine serum albumin (BSA) in a dose-dependent manner [28], whereas no major conformational change was recorded for BSA when adsorbed to carbon C60 fullerene NP [29]. Titanium dioxide (TiO^2^) NP were shown to cause conformational change and reduce polymerization of tubulin, which is an essential cytoskeletal protein [30]. Spectroscopic investigation of zinc oxide (ZnO-NP) interaction with BSA also showed no structural perturbation to its overall structure, however, minor conformational changes were reported [31]. An irreversible conformational change in the secondary structure of the protein transferrin was observed upon interaction with SPIONs [32]. NP-induced protein conformational changes may affect the downstream protein-protein interactions, cellular signalling and also DNA transcription, which is particularly important for enzymes. Loss of enzyme activity can result due to the conformational changes in the active site, brought about by the NP surface. SWCNTs were able to differentially induce loss of structure and catalytic activity for two enzymes investigated [6]. Turci et al. showed that RNAse and lysozyme retained their native structures on silica NP while albumin and lactoperoxidase underwent an irreversible conformational change [33]. Likewise, such conformational changes can also increase the accessibility of the enzyme active site for its substrate. Silica NP were able to induce a molten globule-like conformational change in human carbonic anhydrase while removal of the NP resulted in formation of three intermediate native-like conformations each one of which retained catalytic activity. Covalently bound enzymes horse radish peroxidise, subtilisin Carlsberg, and chicken egg white lysozyme on SWCNTs were also shown to retain their activity and their native structure even under denaturing conditions[34].

**Figure 1:**
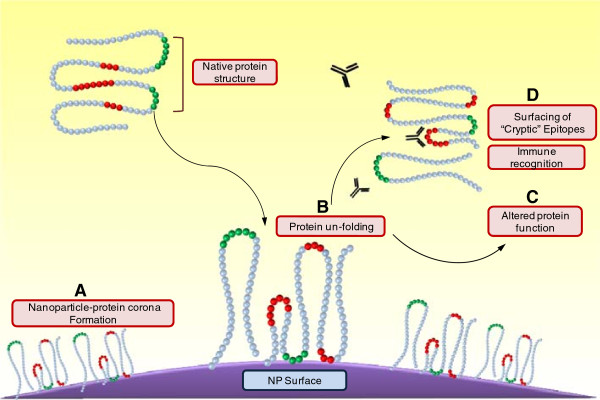
**Schematic representation of NP surface induced unfolding of the interacting protein molecule and consequences.** (**A**) Protein molecules adsorb on to the NP surface, to form a complex termed as the (**B**) NP-PC.NP surface may induce conformational change to the native structure of the adsorbed protein molecule, causing it to unfold. Such protein conformational changesmay either (**C**) alter the function of the native protein moleculeor even lead to (**D**) exposure of “cryptic” epitopes which may result in immunological recognition of the complex.

Conformational epitopes are a result of specific folding of the protein polypeptide chain. Whereas continuous epitopes are regions on the protein primary structure which consist of 10–12 amino acids and are also capable of eliciting an immune response. The NP surface may induce abnormal unfolding of the bound proteins to form novel conformational epitopes or may also induce unfolding of the native protein structure to expose hidden epitopes (Figure 1). Such occult epitopes may affect the functionality of the bound proteins for e.g. elicitation of an unwanted immune response. Deng et al. showed that negatively charged poly (acrylic acid)-conjugated gold NPs bound fibrinogen from blood plasma and induced its unfolding, which in turn activated the receptor Mac-1 on THP-1 cells, causing release of inflammatory cytokines via the NF-κB pathway [35]. Changes in protein structure may lead to loss of tolerance against self, which may in a worst case provoke autoimmune responses and remains an important concern [36].

NPs can also induce conformational changes in proteins that can lead to fibril formation [37,38]. Linse et al. showed that a range of NPs (copolymer, ceria, carbon nanotubes, quantum dots) were capable of inducing fibrillation of β2-microglobulindue to increased protein localization on the NP surface, which led to oligomer formation [39]. Fibrillation of proteins is associated with diseases such as Parkinson’s and Alzheimer’s. The fact that NPs can act as platforms to initiate such protein structural changes demands further investigation of this phenomenon.

The NP surface can also introduce thermodynamic instability to the adsorbed protein molecule making it susceptible to chemical denaturation. ZnO NPs induced unfolding of the periplasmic domain of the ToxR protein of *Vibrio cholera* making the protein susceptible to denaturation by chaotropic agents [40]. Interestingly, ZnO NPs were able to stabilize the α-helical content of lysozyme against denaturing agents [41]. The fate of proteins after binding on the NP surface is thus partially governed by their own chemical properties. A comprehensive list of structural modifications induced by interacting NPs with single proteins has been provided in Table 2.

**Table 2:** Summary of literature on proteins subjected to conformational changes upon interaction with nanoparticle surfaces

**NP type and size**	**Protein investigated**	**Protein mol. wt. and size nm (if provided)**	**Change in protein structure**	**Analytical technique**	**Observations**	**Ref**
ZnO NPs (25 nm)	Vibrio cholera Tox r	32.5 kDa	Yes	CD	NP-protein complex susceptible to denaturation	[40]
ZnO NPs (N/A)	BSA	66 kDa	Yes	CD	Minor conformational changes, secondary structure retained	[31]
ZnO NPs (N/A)	BSA	66 kDa	Yes	FTIR	Minor conformational changes in secondary structure	[42]
TiO_2_ NPs (20 nm)	Tubulin	55kda	Yes	FS	Protein polymerization affected	[30]
SiO_2_ NPs (~40 nm)	BSA	66 kDa	Yes	RS	BSA and lactoperoxidase bound irreversibly	[33]
	Hen egg lysozyme	14.3 kDa	No			
	RNASe A	13.7 kDa	No			
	Lactoperoxidase	77.5 kDa	Yes			
SiO_2_ NPs (6,9,15 nm)	Human Carbonic anhydrase	29 kDa	Yes	NMR	Protein activity was retained	[4]
Alumina and hydroxyapatite particles (100-300 nm)	BSA	66 kDa 8 × 8 × 3	Yes	FTIR	Loss in α-helical structure	[43]
	Hen egg lysozyme	14.3 kDa 4.6 × 3 × 3	Yes			
	Bovine serum fibrinogen	350 kDa 6 × 6.5 × 45	Yes			
Gold (45 nm)	BSA	66 kDa	Yes	CD	Conformational change was dose dependent	[28]
Gold (5-100 nm)	Albumin	67 kDa	Yes	CD and FS	Minor conformational changes observed	[27]
	Fibrinogen	340 kDa	Yes			
	ɣ-globulin	120 kDa	Yes			
	Histone H3	15 kDa	Yes			
	Insulin	5.8 kDa	Yes			
Gold (7-22 nm)	Human Fibrinogen	340 kDa 45 × 5	Yes	CD	Unfolding induced immune response in THP-1 cells	[35]
SPIONs (5-10 nm)	Transferrin	80 kDa	Yes	CD	Irreversible interaction	[32]
SWCNTs (N/A)	Horse radish peroxidase	44 kDa	No	CD	NP-protein complexes retained enzymatic activity	[34]
	Subtilisin Carlsberg	39 kDa	No			
	Chicken egg white lysozyme	14.3 kDa	No			

(Abbreviations used: *CD* Circular dichroism spectrometry, *FTIR* Fourier transformed infrared spectrometry, *FS* Fluorescence spectroscopy, *RS* Raman spectroscopy, *NMR* Nuclear magnetic resonance).

### Nanoparticle-protein corona: implication on cellular interactions

**Figure 2:**
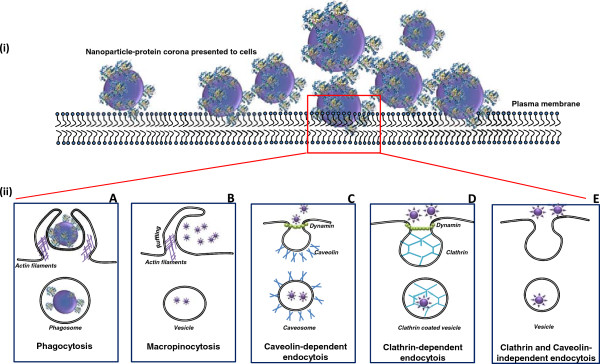
**Interaction of nanoparticles with the cellular interface.** NPs interact with cells via the protein corona. (**A**) Uptake of large sized NP-protein complexes, agglomerates of NP may be ingested by specialized cells such as macrophages and neutrophils via phagocytosis. It involves folding of the plasma membrane over the NP complex to form the phagosome. (**B**) Non-specific uptake of extracellular fluid containing aggregates of NP may also be taken up by cells via macropinocytosis which involves ruffling of the plasma membrane to form vesicles which ultimately fuse to form lysosomes. Endocytosis of NP complexes may also be directed by specific receptors involving formation of (**C**) caveolae that are plasma membrane indentations consisting of cholesterol binding proteins called caveolins or (**D**) clathrin-coated vesicles. (**E**) Apart from these other endocytic mechanisms, independent of clathrin or caveolae may also facilitate uptake of NP.

Given the small size of NPs, it is quite likely that they can encounter different types of cells and also translocate across membrane barriers in an organism. NPs less than 100 nm in diameter can enter cells, less than 40 nm can enter the cell nucleus and below35 nm can cross the blood–brain barrier [44,45]. Uptake of NP can occur via phagocytosis, macropinocytosis or endocytosis (Figure 2 ii). Once taken up, NPs can accumulate in the lysosomes [46,47], intracellular vacuoles as reported in the case of SWCNT uptake by HeLa cells [48], or cytoplasm of cells as observed for copolymer NP [49]. Cytotoxicity and immune-modulation are the two most important repercussions of NP uptake by cells. This is of particular importance when considering NPs that have a propensity to dissolve after reaching the acidic lysosomal compartments of the cell, thus contributing to cellular toxicity. To understand the fate of NPs in the biological context it is imperative to systematically analyse the intricate factors involved in uptake of these novel materials.

Protein adsorption, physical characteristics of the NPs and the properties of interacting cells may influence NP uptake. Kinetics of uptake of the same nanomaterial has been shown to differ with different cell types [50,51]. Adsorption of proteins on the NP surface can take place almost instantly. Therefore, it can be assumed that interaction of the NP with cellular structures is indirect and occurs mostly via the NP-PC and not the bare NP surface (Figure 2 i). The NP-PC can thus influence the uptake of the NP by the cell. The uptake might be either inhibited due to loss of protein structure of an adsorbed protein, or facilitated due to unfolding of the adsorbed protein to access receptors on the cell surface. This is particularly important when looking at differential binding of physiologically active proteins on the NP. Several *in vitro* studies have explored cellular uptake of NP in the presence of serum proteins. Dutta et al. carried out an elaborate study to show that albumin adsorbed on the surface on SWCNTs was responsible for inducing an anti-inflammatory pathway in RAW macrophages, thus highlighting that the identity of the adsorbed protein may dictate the bio-reactivity of the NP surface [52]. Similarly, adsorption of the lung protein SP-A on magnetite NPs was also shown to enhance their uptake by macrophages when compared to ones pre-coated with BSA [53]. Caveolae mediated endocytosis of fluorescent polystyrene NPs (20-100 nm) was shown to be dependent on the presence of albumin on the NP surface. Additionally the caveolae that are cell membrane invaginations typically 60-80 nm in diameter were also shown to contain up to three 20 nm and 240 nm polystyrene NPs, which suggested that these structures can be flexible in accommodating larger sized NP-protein complexes [54].

Apolipoproteins are a class of proteins that are often found in the NP-PC in blood for a number of NP surfaces (Table 1). These have been of interest because of their ability to aid in uptake of NPs by binding to specific receptors on cells. Apolipoproteins B and E were shown to assist in transport of drug bound-polysorbate 80 coated NPs across the blood brain barrier. Receptor mediated endocytosis was speculated to be the means of uptake in this case [55,56]. The impermeable nature of the blood brain barrier makes it difficult to deliver essential drugs and other compounds to the brain. While the ability of NPs to translocate across this barrier provides a promising future in this direction, it also raises important safety concerns regarding toxicity of nanomaterials.

Certain serum proteins such as immunoglobulins and complement pathway proteins possess opsonising characteristics. The presence or enrichment of such opsonising proteins on the NP surface in blood can lead to immune recognition of the NP-protein complex, otherwise not intended. In one study, uptake of NH_2_-polystyrene NPs by macrophages in a protein free medium was shown to change from clathrin-mediated endocytosis to phagocytosis when incubated in serum enriched media [57]. Thus showing that opsonisation of the NP surface by serum proteins remarkably influences its uptake. Adsorption of complement protein C3 and opsonising protein IgG on 50 nm lecithin-coated polystyrene NPs was also shown to increase with time and this directly influenced their uptake by murine Kupffer cells [58].

The large surface area adsorption of proteins on the NP surface often leads to an increase in the hydrodynamic size of the latter. Such large NP-protein complexes can be taken up by phagocytic cell types and also non-phagocytic cells. Lesniak et al. showed that uptake of polystyrene NP by non-phagocytic lung epithelial cells was significantly higher when incubated in non-heated serum compared to NPs incubated in heat inactivated serum [59]. While this study does not state the exact mechanism of uptake the authors report that the amount of protein and also the presence of heat labile complement proteins in the non-heat inactivated serum may be responsible for the observed enhanced uptake.

Another possible explanation for the enhanced uptake is that, cellular interaction of the NP is non-specific; depending exclusively on the amount of protein, rather than the presence of certain proteins on the NP surface as discussed above. This was shown by Ehrenberg et al. in their study where, NPs incubated with complete serum or serum depleted of several abundant proteins did not affect the association of NPs with endothelial cells *in vitro*[16]. While it is interesting to know that protein binding on the NP surface facilitates its uptake and other interactions, there have been some contradictory reports in the literature. Uptake of FePt NPs by HeLa cells was suppressed in the presence of the NP-PC [23]. Also, silica NPs dispersed in serum free medium were taken up more efficiently by lung epithelial cells as compared to ones in presence of 10% serum. By and large, the amount and identity of the protein adsorbed on the NP surface seems to determine the uptake of the NP.

Apart from protein adsorption other minor differences in physicochemical characteristics such as zeta potential, and size of the NP surface have also been shown to influence their mode of uptake by cells [51,60-63]. Interestingly, sedimentation capacity was proposed to be the driving factor for uptake of gold NP by human breast cells. This was clearly demonstrated by exposing human breast cancer cells to gold NPs in upright or inverted configurations [64]. Also, Kim and co-workers reported that the phase of cell cycle was an important factor that influenced uptake of 40 nm yellow-green PS-COOH by A549 cells. They also demonstrated that the NP taken up by the parent cells were divided between the daughter cells upon cell division suggesting implications on clearance or accumulation of NPs *in vivo*[65].

A review of the literature pertaining to cellular uptake of NPs reveals a number of inconsistencies regarding factors influencing this interaction. It has to be taken into account that most of these studies are conducted under *in vitro* conditions, often with immortal cell lines which may show different characteristics as compared to their *ex vivo* counterparts. Moreover, *in vivo* NPs interact not only with the protein micro-environments but also other cellular moieties simultaneously, making it a challenging task to correctly extrapolate behaviour of NPs *in vivo.* Perturbation of the native structure of the bound protein depends on the surface of the interacting NP and together these two factors direct the bio-reactivity of the NP.

### Physico-chemical characteristics of the nanoparticle surface influence protein adsorption and therefore cellular interactions

NP composition [66,67], hydrophobicity [33], presence of specific functional groups, pH and temperature [68] have been shown to affect protein adsorption on the surface of NPs. Sedimentation of NPs especially in an *in vitro* exposure system has also been reported to influence cellular interactions [64].

Colloidal solutions of NPs often have a tendency to form agglomerates. NP size, concentration, and surface charge can influence agglomerate formation. Coarse NP agglomerates can exert noticeably different biological properties compared to NPs that are efficiently dispersed [69-71]. Agglomeration can also change the available surface area for protein binding. Uneven surface of the agglomerated NP can induce protein conformational changes. NPs dispersed in protein free media often tend to agglomerate. Studies have reported the use of ultrasonic energy, surfactants [72], polymer coating [73] protein containing dispersion media like serum, alveolar lung fluid, etc. to control agglomeration [52,74-77]. Dispersal of high concentrations of NPs in solutions containing certain proteins such as fibrinogen can in contrast lead to aggregation, due to the formation of inter-particle bridges by the protein [78,79].

Yet another physical characteristic of the NP known to influence protein binding is size. Twelve nm sized negatively charged poly acrylic acid Au NPs were reported to bind fibrinogen with higher affinity compared to 7 nm NPs [79]. In another study, 15 nm silica NPs induced a six-fold higher change in the secondary structure of human carbonic anhydrase I protein compared to 6 nm silica NPs [4]. Stability of ribonuclease A was dramatically reduced with binding to silica NPs of increasing sizes [80]. Silica NPs (100 nm) were capable of inducing greater loss of structure and function for the protein lysozyme as compared to 4 nm sized particles [81]. On the other hand, Dutta et al. reported that plasma protein adsorption profiles remained uniform for differentially sized silica NPs [52]. This emphasizes again, that it is very difficult to formulate rules about protein interactions that apply to different types of NPs.

The shape of the NP can be important as well. This was confirmed for TiO_2_ nanorods and nanotubes that differentially adsorbed plasma proteins [21]. Likewise, modification of NP surface charge can also influence protein adsorption. Studies have demonstrated that NP surfaces with no charge bind less proteins than their negatively charged (COOH functionalized) or positively charged (NH_2_ functionalized) counterparts [18,82]. Polyethyleneimine-functionalization of (ZnO) NPs favoured their interaction with albumin as compared to pristine ones [47]. A recent study showed that structural modification of a self-protein such as fibrinogen can be affected by the surface properties of gold NPs. Negatively charged gold NPs, unlike positively or neutral charged ones, were shown to bind fibrinogen in an orientation that led to cytokine release in human monocytic THP-1cells *in vitro*[18]. Influence of the NP surface on immune stimulation by a self- protein is an important concern. Application of this information to extrapolate adverse effects of such interactions under *in vivo* conditions however needs further study.

Chemical fabrication of the NP surface to avoid adsorption of proteins can be carried out using polyethylene glycol (PEG), also referred to as “PEGylation”. This imparts a “stealth character” to an NP surface, shielding it from being recognized by immune cells [83]. NPs can be made to remain in circulation for longer periods of time by controlling the density of PEG on their surface [83,84]. Siliconate treatment of the NP surface too has been reported to prevent protein adsorption [21].

An important issue that requires further attention is the indirect influence of the NP-physico-chemical characteristics on cytotoxicity, cell signalling etc. Cytotoxicity and cytokine release by lung epithelial cells when exposed to ZnO or TiO_2_nano-powders was influenced by their shape and crystalline forms, respectively [85]. Differential inflammatory potential and cellular association was recorded for spherically shaped or sheets of nano ZnO [86]. Silver nanorods were shown to be toxic to the human lung epithelial cells, while nanospheres with the same mass concentration were not [87]. Thus, the influence of the proteins adsorbed on the NP surface cannot be disregarded completely when assessing immunotoxic outcomes of NPs.

### Analytical approaches to study nanoparticle-protein interactions

**Figure 3:**
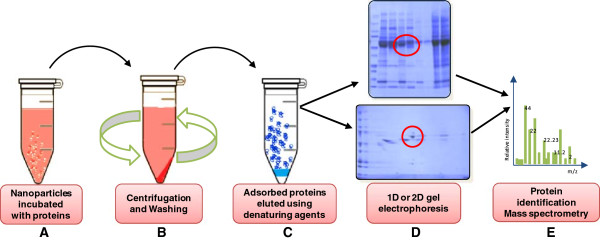
**Schematic representation of the commonly used strategy to isolate and identify surface adsorbed proteins, when nanoparticles interact with complex protein mixtures.** (**A**) Incubation of NP with protein solutions results in adsorption of protein onto the NP surface. Protein concentration may affect the amount and identity of proteins adsorbed on the presented NP surface. (**B**) Centrifugation for removal of unbound proteins followed by repeated washing of the NP-protein pellet is important for isolation of the “hard protein corona”. (**C**) Isolation of the NP-PC can be achieved by elution of the adsorbed proteins using denaturing agents such as Laemmli buffer which contains sodium dodecyl sulphate and 2-mercaptoethanol that facilitate the overall desorption of the proteins. (**D**) The desorbed proteins can thus be separated using one or two dimensional gel electrophoresis. (**E**) Separated protein bands of interest can further be subjected to tryptic digestion and can be subsequently identified using mass spectrometric methods.

Isolation and identification of proteins constituting the NP-PC are imperative to understand bio-reactivity of NPs. Interaction of NP surfaces with individual proteins or proteomes can be studied using a range of analytical techniques (Table 3). Mass spectrometry (MS) based proteomics is the most widely used strategy to study the NP-PC (Figure 3). Despite the qualitative nature of the outcomes of this technique, it can be applied over a range of NPs, which makes it a preferred choice. A useful development to MS based proteomics is the use of stable isotope labelling by amino acids in cell culture (SILAC). This technique has been used to not only identify but also quantify amounts of proteins bound on NP surfaces [88]. Interactions of NP surfaces with single purified proteins can be investigated by fluorescence spectroscopy and circular dichroism (CD) spectroscopy. Fluorescence spectroscopy makes use of the intrinsic fluorescence of the protein whereas CD spectroscopy uses changes in chiral properties of the protein to predict changes in its secondary structure. High resolution information on protein structure can also be provided by nuclear magnetic resonance (NMR) spectroscopy. Specific interactions of ubiquitin molecules with gold NP surface was studied using NMR, which made it possible to identify the exact ubiquitin domain that bound to the NP surface [89]. Fluorescent correlation spectroscopy has also been used to monitor protein adsorption on NP surfaces and has been shown to be sensitive at nanomolar quantities of nanoparticles.

**Table 3:** Summary of analytical techniques to conduct physico-chemical characterisation, monitor nanoparticle surface driven protein conformational changes and uptake of nanoparticles by cellular structures

**Analysis of**	**Analytical technique**	**Brief description**	**Ref**
**Nanoparticle physical characterisation**			
Size and charge	Dynamic light scattering	Changes in the hydrodynamic diameter of NP upon binding to proteins	[1]
	Analytical Ultracentrifugation	Changes in the hydrodynamic diameter of NP	[6]
Dissolution	Inductively coupled mass spectrometry	For detecting elemental composition of the nanomaterial	[90]
Shape and structure	X ray diffraction	Determination of crystalline structure	[38]
	Electron microscopy	Visualisation of nanoparticle structure	
Surface area	Braunauer Emmet Teller method	Measures specific surface area using adsorption of gas on the surface	[38]
De-agglomeration	Ultrasonication	Uses sound energy to disrupt large aggregates of NP	[36]
**Nanoparticle protein interaction**			
Protein binding affinity	Isothermal calorimetry	To measure binding constant, thermodynamic parameters of NP-protein interactions	[2]
	Fluorescence spectroscopy	Measures change in fluorescence spectra due to NP-protein interaction	[65]
	UV–vis spectroscopy	Measures change in absorption spectra due to NP-protein interaction	[57]
	Quartz crystal balance	Detects change in mass at the oscillating quartz surface due to NP-protein interaction	[91]
	Surface Plasmon resonance	Detects change in oscillation of electrons on a metal surface due to NP-protein interaction	[92]
	Atomic force microscopy	Gives surface profile of the nanomaterial	[93]
	Fluorescence correlation spectroscopy	Binding characteristics depending on fluctuation in florescence	[94]
**Nanoparticle surface induced protein structure changes**			
Protein structural changes after binding	Circular Dichroism spectroscopy	Measures changes in secondary structure of proteins depending on chiral properties of proteins	[61]
	Fourier transformed infrared spectroscopy	Measures adsorption of amide bonds in the proteins to derive structural change	[43]
	Raman spectroscopy	Studies molecular vibrations to predict structure	[52]
	Nuclear Magnetic Resonance	Relies on magnetic properties of atomic nuclei to predict structure	[4]
**Nanoparticle- Cellular interactions**			
NP uptake	Confocal microscopy	Visualization of fluorescent nanoparticles *in vitro*	[59]
	Confocal micro Raman spectroscopy		[95]

The use of other techniques such as isothermal titration calorimetry, surface plasmon resonance has also been reported to characterise protein binding to various surfaces. A recent study showed that 70 nm copolymer NPs bind around 650 human serum albuminmolecules and 200 nm particles can accommodate up to 4600 molecules, using isothermal calorimetry [2]. Quartz crystal microbalance, a technique that detects change in mass at the oscillating quartz surface due to NP-protein interaction, was employed to probe adsorption of myoglobin, BSA, and cytochrome c proteins on the surface modified gold NPs [91]. The choice of analytical techniques for studying NP-PC greatly depends on the physical state of the NP.

Visualization of NPs uptake by cells is often carried out by florescent labelling of the NP surface or synthesis of fluorescent NPs which can be detected by flow cytometry or confocal laser scanning microscopy. Fluorescent labelling of NPs can however modify its surface thus interfering with subsequent protein interactions. Confocal Raman microscopy was recently used to study uptake of Al_2_O_3_ and CeO_2_ NPs by Lopis and co-workers [95]. This technique was reported to be label-free and specific at the single cell level.

Studies have attempted to propose models to generalize different aspects of NP-protein interactions. Dell’Orco and co-workers formulated a mathematical model to predict kinetics of protein binding to NP surfaces that can be extended to any proteome-NP combination [96]. Nangi et al. developed a simulation model to predict energy barriers, translocation rate constants and half-lives of NPs through lipid membranes as a function of their physical properties [97]. Xia et al. introduced the biological surface adsorption index (BSAI) to predict the order in which factors such as hydrophobicity, hydrogen bonding, polarizability of NP surface and ion-pair electrons affect protein adsorption at the nano-bio interface, using12 physico-chemically defined protein-mimicking probes and MWCNTs [98]. The experimental approach employed by most studies currently involves detailed study of a single protein with the NP surface rather than the complete NP-PC that consists of several different types of proteins. Strategic use and combination of the available analytical techniques is needed to analyse several aspects of NP-protein interactions simultaneously.

## Conclusion

Characterization and analysis of proteins bound to the NP surface is the first step towards understanding the true nature of the NP-mediated biological effects. Research thus far highlights that size, shape, and surface characteristics of NPs affect protein adsorption and also have the capability to modify the structure of the adsorbed protein molecules. This can significantly affect the reactivity of the NP with cells and determine the route and efficiency of NP uptake. The adsorbed proteins may also promote translocation of the NP across cellular barriers, and clearance or accumulation in vital organs. Interestingly, most studies conducted in this direction focus on *in vitro* test systems, therefore, extrapolation of this information in predicting behaviour of NPs *in vivo* remains a challenging task and needs further investigation. Systematic analysis of binding characteristics of novel NPs with proteins having different structures, shapes and functional properties can enhance our existing knowledge about NP-protein interactions. Thorough understanding of NP-protein interactions might lead to strategic manipulation of NP surfaces to adsorb specific functional proteins or small drug molecules intended for delivery *in vivo*. Furthermore this knowledge might also prove to be useful in predicting nanotoxicity related safety concerns. In summary, NP-PC dictates the overall biological reactivity of the otherwise inorganic NP surface. Understanding the dynamics of this complex interaction can thus provide useful insights into cytotoxic, inflammatory potential and other key properties of these novel materials that can be explored for developing safer and value added nanomaterials for future applications.

## Abbreviations

NP: Nanoparticles; NP-PC: Nanoparticle-protein corona; SWCNT: Single walled carbon nanotubes; SPION: Superparamagnetic iron oxide nanoparticles; MWCNT: multi walled carbon nanotubes; TiO2: Titanium dioxide; ZnO: Zinc oxide; PEG: Polyethylene glycol; BSA: Bovine serum albumin; FePt-NP: Iron-Platinum Nanoparticles; BSAI: Biological surface adsorption index; CD: Circular dichroism spectrometry; FTIR: Fourier transformed infrared spectrometry; FS: Fluorescence spectroscopy; RS: Raman spectroscopy; NMR: Nuclear magnetic resonance.

## Competing interests

The authors declare that they have no competing interests.

## Authors’ contributions

S.S. has conducted the literature review and written the first draft of the manuscript. A.D. and A.L. and provided advice on the theme of the manuscript and also helped to edit, revise and provided final approval of the manuscript. All authors read and approved the final manuscript.
